# High antimicrobial resistance in urinary tract infections in male outpatients in routine laboratory data, Germany, 2015 to 2020

**DOI:** 10.2807/1560-7917.ES.2022.27.30.2101012

**Published:** 2022-07-28

**Authors:** Jonas Salm, Florian Salm, Patricia Arendarski, Tobias Siegfried Kramer

**Affiliations:** 1 Charité – Universitätsmedizin Berlin, corporate member of Freie Universität Berlin and Humboldt-Universität zu Berlin, Berlin School of Public Health, Berlin, Germany; 2 Institute of Medical Microbiology and Hygiene, Medical Centre – University of Freiburg, Faculty of Medicine, University of Freiburg, Freiburg, Germany; 3 Prevent Infect, Bad Krozingen, Germany; 4 LADR Laboratory Group Dr Kramer & Colleagues, Geesthacht, Germany; 5 Charité – Universitätsmedizin Berlin, corporate member of Freie Universität Berlin and Humboldt-Universität zu Berlin, Institute for Hygiene and Environmental Medicine, Berlin, Germany

**Keywords:** Urinary tract Infections, antimicrobial resistance, Germany, outpatient, ambulatory, antimicrobial stewardship

## Abstract

**Background:**

Evidence on the distribution of bacteria and therapy recommendations in male outpatients with urinary tract infections (UTI) remains insufficient.

**Aim:**

We aimed to report frequency distributions and antimicrobial resistance (AMR) of bacteria causing UTI in men and to identify risk factors for resistance of *Escherichia coli* against trimethoprim (TMP) and ciprofloxacin (CIP).

**Methods:**

We conducted a retrospective observational study using routinely collected midstream urine specimens from 102,736 adult male outpatients sent from 6,749 outpatient practices to nine collaborating laboratories from all major regions in Germany between 2015 and 2020. Resistance in *E. coli* was predicted using logistic regression.

**Results:**

The three most frequent bacteria were *E. coli* (38.4%), *Enterococcus faecalis* (16.5%) and *Proteus mirabilis* (9.3%). Resistance of *E. coli* against amoxicillin (45.7%), TMP (26.6%) and CIP (19.8%) was common. Multiple drug resistance was high (22.9%). Resistance against fosfomycin (0.9%) and nitrofurantoin (1.9%) was low. Resistance of *En. faecalis* against CIP was high (29.3%). Isolates of *P. mirabilis* revealed high resistance against TMP (41.3%) and CIP (16.6%). The CIP and TMP resistance was significantly higher among bacteria derived from recurrent UTI (p < 0.05). Age ≥ 90 years, recurrent UTI and regions East and South were independently associated with AMR of *E. coli* against TMP and CIP (p < 0.05).

**Conclusion:**

The most frequent UTI-causing pathogens showed high resistance against TMP and CIP, empirical therapy is therefore likely to fail. Apart from intrinsically resistant pathogens, susceptibility to fosfomycin and nitrofurantoin remains sufficient. Therefore, they remain an additional option for empirical treatment of uncomplicated UTI in men.

## Introduction

Urinary tract infections (UTI) are among the most common community-acquired infections in humans [1]. For this and other reasons, UTI are among the most common causes for antibiotic prescription [2].

In outpatients, UTI occur predominantly as an acute uncomplicated cystitis [3]. According to the German guideline for acute UTI, patients are divided into five groups regarding diagnostics as well as therapeutic recommendations [4]. As UTI predominantly occur in young female patients [5], this patient group has been investigated most and therefore, empirical therapy as well as diagnostic recommendations are evidence-based in this group [4].

Nevertheless, UTI can also occur in male patients [6]. Even though male patients can have an uncomplicated UTI, they are most commonly categorised as complicated [4,7]. Around 20% of males will be affected by an UTI in their lifetime [8], whereas around 10–15% of females have an uncomplicated UTI each year [9]. Considering this difference in frequency, it is not surprising that there is little evidence for empirical therapy recommendations among men with UTI [4,6]. International and national guidelines recommend treatment with fluoroquinolones or trimethoprim/sulfamethoxazole (SXT). If a prostatitis can be excluded, treatment with nitrofurantoin or pivmecillinam can be considered [4,10]. The latter two have been discussed as highly effective empirical treatment options [11,12]. Unfortunately, bacterial prostatitis is difficult to exclude clinically and difficult to treat. Only few antibiotics achieve sufficient tissue concentrations and resistance against antibiotics with a high oral bioavailability, such as ciprofloxacin (CIP), is common. Despite these difficulties, fosfomycin has been discussed as a promising alternative for first-line oral therapy of bacterial prostatitis [13,14].

A closer look into incidences of UTI reveals pronounced differences by age. In younger patients, UTI occur predominantly in females [9], whereas the incidence in male and female patients 85 years and older is comparable with respectively 7.8 and 12.8 per 100 person-years [15,16]. This underlines once more the importance of evidence-based recommendations for male patients.

According to the German national guideline, urine cultures before start of the antibiotic therapy are recommended in male patients with suspected UTI [4]. Therefore, routine laboratory data are available which could generate insights into pathogen as well as antimicrobial resistance (AMR) patterns. These data could inform empirical antimicrobial therapy and be used to document the development of AMR over time among male outpatients with UTI.

This research investigated urine cultures of male outpatients in Germany. We analysed the distribution of bacteria as well as the resistance of the three most common bacteria against frequently used oral antibiotics. A secondary aim was to identify risk factors for the occurrence of AMR of *Escherichia coli* isolates by patient characteristics.

## Methods

### Study design

We retrospectively analysed data derived from routine urine cultures of male outpatients from 2015 to 2020. Urine culture diagnostics and data storage were performed by medical laboratories of the private LADR Laboratory Group Dr Kramer and Colleagues. We included data from nine of their laboratories in the north, east, south and west of Germany (for further information see Supplementary Figure S1 on laboratory sites of participating laboratories). The datasets were extracted using the hygiene management system HyBASE and stored as pseudonymised Excel files.

To ensure that the dataset only contained positive and relevant urine cultures, we extracted the data on those bacteria known to be relevant for causing UTI according to the Quality Standards for the Microbiological Diagnosis of Infectious Diseases (Mikrobiologisch-infektiologische Qualitätsstandards; MIQ) [17]. Further, we excluded all cultures which were not from midstream urine and with a bacteria count lower than 10^4^ colony-forming units per mL. In a next step we excluded every result with a positive test of growth inhibition of *Bacillus simplex*. After applying these inclusion and exclusion criteria, the remaining urine culture results were defined as representing the clinical diagnosis UTI.

We generated the binary variables 'recurrent UTI' and 'polymicrobial'. According to the German guideline, we labelled every positive urine culture in the same patient which occurred within 6 months since the last positive testing as recurrent [4]. Every positive result after these 6 months was defined as a new infection. We did not consider the first 7 days after the initial urine culture for the definition of recurrence, to account for multiple testing of the same initial UTI. Urine culture results were labelled as polymicrobial if one patient had multiple urine culture results on the same date with different relevant pathogen isolates.

### Study participants

We included in the study all male outpatients ≥ 18 years with midstream urine cultures within the study period. These samples were sent by 6,749 outpatient practices in Germany. For every study participant, we had complete urine culture results of relevant bacteria which are known to cause UTI. Since one patient could be infected with more than one bacterium, there are fewer patients than pathogen isolates. 

### Microbiology

Pathogen identification and antimicrobial susceptibility testing (AST) was performed with automated systems such as MALDI-TOF, Vitek2, disc diffusion and microbroth dilution. We included susceptibility testing against: fosfomycin, nitrofurantoin, nitroxoline, mecillinam, trimethoprim (TMP), trimethoprim/sulfamethoxazole (SXT), ciprofloxacin (CIP), amoxicillin, amoxicillin-clavulanic acid (AMC), cefuroxime, cefpodoxime and vancomycin. The results were interpreted according to the European Committee on Antimicrobial Susceptibility Testing (EUCAST) [18] and the German National Committee for sensitivity testing of antibiotics (Nationales Antibiotika-Sensitivitätstest-Komitee; NAK) [19]. To test for multiple drug resistance, we analysed cross-resistance between CIP, SXT, AMC and cefpodoxime. Multidrug resistance (MDR) was defined as resistance against at least two of those four antibiotics. Extensive drug resistance (XDR) was defined as resistance against at least three of these antibiotics. Pandrug resistance (PDR) was defined as resistance against all four antibiotics.

### Statistical analysis

Descriptive analyses for patient characteristics are reported as means with standard deviation (SD) for continuous variables and as counts with percentages for categorical variables. Antimicrobial susceptibility of a species is reported as the percentage of resistant isolates among all tested isolates. We calculated 95% confidence intervals (CI) for proportions using the Clopper–Pearson method. Logistic regression was performed to identify risk factors for AMR in *E. coli*. As binary outcome variable we used AMR. The selection of predictor variables was done a priori using subject matter knowledge. The results of the logistic regression models are reported as odds ratios (OR) with 95% CI and corresponding p values. The evaluation of the AST per pathogen as well as logistic regression modelling were performed as complete case analyses. Differences in AMR were considered as statistically significant if the corresponding 95% CI did not overlap [20]. The assignment to region, to detect differences in AMR, was based on the locations of laboratories. All statistical analyses were performed using the free software for statistical computing and graphics R (R 4.0.3; R Foundation, Vienna, Austria). The significance level was set to α = 0.05.

## Results

### Study sample

Overall, 210,178 male patients from 6,749 outpatient practices in Germany were included into the study. After applying inclusion and exclusion criteria, we analysed urine culture results of 102,736 male patients (Supplementary Figure S2 provides a visual summary of the data processing steps). The mean age of study participants was 69.3 years (SD: ± 14.93). In total 25,249 patients (24.6%) were considered as having a recurrent UTI. Patients with recurrent UTI had a mean age of 71.5 years (SD: ± 14.00) (Table 1).

**Table 1:** Characteristics of study participants stratified by recurrence, urinary tract infections in male outpatients, Germany, 2015–2020 (n = 102,736)

	Overall (n = 102,736)		Non-recurrent UTI (n = 77,487)		Recurrent^a^ UTI (n = 25,249)	
	n	%	n	%	n	%
**Age in years**						
Mean (SD)	69.3 (14.93)		68.6 (15.16)		71.5 (14.00)	
**Age groups (years)**						
18–29	2,280	2.2	1,900	2.5	380	1.5
30–39	3,140	3.1	2,559	3.3	581	2.3
40–49	5,378	5.2	4,323	5.6	1,055	4.2
50–59	12,585	12.3	10,043	13.0	2,542	10.1
60–69	20,011	19.5	15,498	20.0	4,513	17.9
70–79	30,579	29.8	22,788	29.4	7,791	30.9
80–89	26,186	25.5	18,527	23.9	7,659	30.3
≥ 90	2,577	2.5	1,849	2.4	728	2.9
**Culture**						
Polymicrobial	24,894	24.2	17,414	22.5	7,480	29.6
**Region**						
North	36,824	35.8	27,912	36.0	8,912	35.3
East	12,476	12.1	9,133	11.8	3,343	13.2
South	12,928	12.6	9,853	12.7	3,075	12.2
West	40,508	39.4	30,589	39.5	9,919	39.3

UTI: urinary tract infection.

^a^ More than one positive urine culture in the past 6 month.

### Pathogen distribution

In the study sample of 102,736 male patients, 131,498 bacterial isolates were detected. In total, 34,000 (25.9%) of these isolates belonged to recurrent UTI, whereas 53,632 (40.8%) belonged to polymicrobial cultures. Table 2 summarises the corresponding frequency distributions of bacterial species. The most frequent pathogen was *E. coli* with 50,505 (38.4%) detected isolates (Table 2). The next most frequent pathogens were *Enterococcus faecalis* and *Proteus mirabilis* with 21,632 (16.5%) and 12,240 (9.3%) isolates, respectively (Table 2). Stratification by mixed infection and by recurrence changed the relative frequencies of pathogens. Especially *En. faecalis* hat a noticeably higher proportion of 22.9% in polymicrobial infections compared with 12.0% in monomicrobial infections (Table 2). The proportion of *E. coli* was lower in recurrent UTI with 33.5% detected and higher in monomicrobial infections with 48.3%, compared with 38.4% in the total sample (Table 2).

**Table 2:** Frequency distribution of bacteria detected in midstream specimens of urine, isolates from male outpatients, Germany, 2015–2020 (n = 131,498)

	Overall (n = 131,498)		Monomicrobial (n = 77,866)		Polymicrobial (n = 53,632)		Non-recurrent UTI (n = 97,498)		Recurrent^a^ UTI (n = 34,000)	
	n	%	n	%	n	%	n	%	n	%
**Pathogen**										
*Escherichia coli*	50,505	38.4	37,596	48.3	12,909	24.1	39,117	40.1	11,388	33.5
*Enterococcus faecalis*	21,632	16.5	9,353	12.0	12,279	22.9	15,908	16.3	5,724	16.8
*Proteus mirabilis*	12,240	9.3	6,407	8.2	5,833	10.9	8,780	9.0	3,460	10.2
*Klebsiella pneumoniae*	10,121	7.7	5,675	7.3	4,446	8.3	6,968	7.2	3,153	9.3
*Pseudomonas aeruginosa*	7,690	5.9	3,390	4.4	4,300	8.0	4,899	5.0	2,791	8.2
*Klebsiella* spp.	5,436	4.1	2,781	3.6	2,655	5.0	4,006	4.1	1,430	4.2
*Citrobacter* spp.	4,985	3.8	2,675	3.4	2,310	4.3	3,766	3.9	1,219	3.6
*Staphylococcus aureus*	4,243	3.2	2,396	3.1	1,847	3.4	2,985	3.1	1,258	3.7
*Enterobacter* spp.	4,074	3.1	2,304	3.0	1,770	3.3	2,950	3.0	1,124	3.3
*Morganella* spp.	2,514	1.9	1,071	1.4	1,443	2.7	1,909	2.0	605	1.8
β-haemolytic Streptococci^b^	2,139	1.6	1,383	1.8	756	1.4	1,809	1.9	330	1.0
Others^c^	5,919	4.5	2,835	3.6	3,084	5.8	4,401	4.5	1,518	4.5

UTI: urinary tract infection.

^a^ More than one positive urine culture within the last 6 month.

^b^ *Streptococcus agalactiae* and *Streptococcus pyogenes.*

^c^ *Enterococcus spp.; Proteus spp.; Providencia spp.; Aerococcus urinae; Aerococcus sanguinicola; Enterococcus faecium; Mycoplasma/Ureaplasma; Corynebacterium urealyticum; Staphylococcus saprophyticus.*

### Antimicrobial susceptibility

Antibiotics recommended for patients with uncomplicated UTI according to the German guideline were labelled as first-line. Reported resistance rates were based on the total number of tested isolates per antibiotic. The reported total AMR was calculated considering only non-recurrent UTI. In total, *E. coli* isolates revealed resistance rates below 3% against all first-line antibiotics except for mecillinam with 8.4% (95% CI: 7.9–8.9) (Table 3). Resistance of *E. coli* against amoxicillin was 45.7% (95% CI: 45.2–46.2). Resistance of *E. coli* against AMC was comparatively lower with 29.9% (95% CI: 29.33–30.45) resistant among all tested isolates.

**Table 3:** Single and multiple antimicrobial resistance in non-recurrent urinary tract infections in male outpatients, Germany, 2015–2020 (n = 53,621)

	*Escherichia coli*			*Enterococcus faecalis*			*Proteus mirabilis*		
	R^a^ in %	95% CI	n tested	R^a^ in %	95% CI	n tested	R^a^ in %	95% CI	n tested
**First-line antibiotics**									
FOF	0.9	0.8–1.0	38,365	ND^b^			12.4	11.7–13.1	8,532
NTX	0.9	0.7–1.2	5,171	ND^b^			2.6	1.8–3.7	1,260
NIT	1.9	1.8–2.1	37,995	0.8	0.6–0.9	13,715	ND^c^		
MEC	8.4	7.9–8.9	12,105	ND^b^			29.3	26.5–32.1	1,063
**Trimethoprim ± sulfamethoxazole**									
TMP^d^	26.6	26.2–27	37,995	ND^e^			41.3	40.1–42.5	6,560
SXT	21.6	21.2–22.1	38,403	ND^e^			36.3	35.2–37.3	8,562
**Fluoroquinolones**									
CIP	19.8	19.4–20.2	38,470	29.3	28.6–30.1	12,970	16.6	15.9–17.4	8,577
**Cephalosporins**									
CXM	12.5	12.1–12.8	32,345	ND^f^			2.8	2.4–3.2	7,338
CPD	11.3	11.0–11.7	36,050	ND^f^			1.5	1.2–1.8	7,930
**Aminopenicillins**									
AMX	45.7	45.2–46.2	38,438	0.2	0.1–0.2	15,272	33.2	32.2–34.2	8,574
AMC	28.2	27.8–28.7	38,353	0.2	0.1–0.2	15,272	9.3	8.7–9.9	8,548
**Glycopeptides**									
VAN	ND			0.1	0–0.1	15,064	ND		
**Multiple drug resistance^g^**									
MDR	22.9	22.5–23.4	35,936	ND^h^			17.8	16.9–18.6	7,898
XDR	11.5	11.1–11.8	35,936	ND^h^			3.8	3.4–4.2	7,898
PDR	4.4	4.2–4.6	35,936	ND^h^			0.7	0.5–0.9	7,898

AMC: amoxicillin-clavulanic acid; AMX: amoxicillin; CI: confidence interval; CIP: ciprofloxacin; CPD: cefpodoxime; CXM: cefuroxime; EUCAST: European Committee on Antimicrobial Susceptibility Testing; FOF: fosfomycin; MDR: multidrug resistance; MEC: mecillinam; MIC: minimum inhibitory concentration; ND: not done; NIT: nitrofurantoin; NTX: nitroxoline; PDR: pandrug resistance; R: resistance rate; SXT: trimethoprim/sulfmethoxazole; TMP: trimethoprim; VAN: vancomycin; XDR: extensive drug resistance.

^a^ According to EUCAST only 'R' was considered as resistant [18].

^b^ No MIC breakpoints according to EUCAST [18].

^c^ MIC breakpoints for *E. coli* were also used for *P. mirabilis.*

^d^ TMP as first-line drug if local antimicrobial resistance < 20%.

^e^ According to EUCAST uncertain activity against enterococci [18].

^f^ Intrinsically resistant.

^g ^Isolates resistant against at least two (MDR), three (XDR) or four (PDR) of the following antibiotics: AMC, CIP, CPD, SXT.

^h^ As only two of the considered antibiotics are active against enterococci the concept of multiple drug resistance was not applied to *En. faecalis.*

Resistance of *En. faecalis* against nitrofurantoin was statistically significantly lower than that of *E. coli* (Table 3). Resistance of *P. mirabilis* against all first-line antibiotics was statistically significantly higher than of *E. coli* (Table 3). In contrast, MDR was statistically significantly less frequent in *P. mirabilis* isolates than in *E. coli.*

Table 4 illustrates the AMR in total and stratified by recurrence. Intrinsically resistant bacteria were counted as resistant. The expected empirical susceptibility was highest for nitrofurantoin (74.9%) and CIP (78.0%) (Table 4).

**Table 4:** A priori total antimicrobial resistance and stratified by recurrence in urinary tract infections in male outpatients, Germany, 2015–2020 (n = 131,498)

	Total			Recurrent UTI			Non-recurrent UTI		
	R^a^ in %	95% CI	n tested	R^a^ in %	95% CI	n tested	R^a^ in %	95% CI	n tested
**First-line antibiotics**									
FOF	35.5	35.2–35.7	127,465	38.6	38.1–39.1	32,938	34.4	34.1–34.7	94,527
NTX	75.6	75.3–76	52,246	75.9	75.2–76.6	14,576	75.5	75.1–76	37,670
NIT	25.1	24.8–25.3	120,512	30.8	30.3–31.4	31,241	23.1	22.8–23.3	89,271
MEC	41.6	41.0–42.1	32,200	46.5	45.4–47.5	9,100	39.7	39.0–40.3	23,100
**Trimethoprim ± sulfamethoxazole**									
TMP^b^	48.5	48.2–48.8	104,285	56.3	55.7–56.9	27,476	45.7	45.3–46.1	76,809
SXT	41.3	41.1–41.6	128,481	48.2	47.6–48.7	33,384	38.9	38.6–39.2	95,097
**Fluoroquinolones**									
CIP	22.0	21.8–22.3	122,323	31.8	31.3–32.3	31,688	18.6	18.4–18.9	90,635
**Cephalosporins**									
CXM	43.5	43.2–43.8	111,030	50.0	49.4–50.6	28,930	41.2	40.8–41.5	82,100
CPD	39.4	39.1–39.7	117,729	45.2	44.7–45.8	30,598	37.3	37.0–37.7	87,131
**Aminopenicillins**									
AMX	52.0	51.8–52.3	120,855	58.1	57.6–58.7	30,985	49.9	49.6–50.3	89,870
AMC	39.5	39.1–39.9	73,271	46.8	46.1–47.5	19,190	36.9	36.5–37.3	54,081

AMC: amoxicillin-clavulanic acid; AMX: amoxicillin; CIP: ciprofloxacin; CPD: cefpodoxime; CXM: cefuroxime; EUCAST: European Committee on Antimicrobial Susceptibility Testing; FOF: fosfomycin; MEC: mecillinam; NIT: nitrofurantoin; NTX: nitroxoline; R: resistance; SXT: trimethoprim/sulfmethoxazole; TMP: trimethoprim; VAN: vancomycin; VAN: vancomycin.

^a ^According to EUCAST [18], only 'R' was considered as resistant, intrinsically resistant bacteria were counted as 'R'.

^b^ TMP as first-line drug if local antimicrobial resistance < 20%.

**Figure 1:**
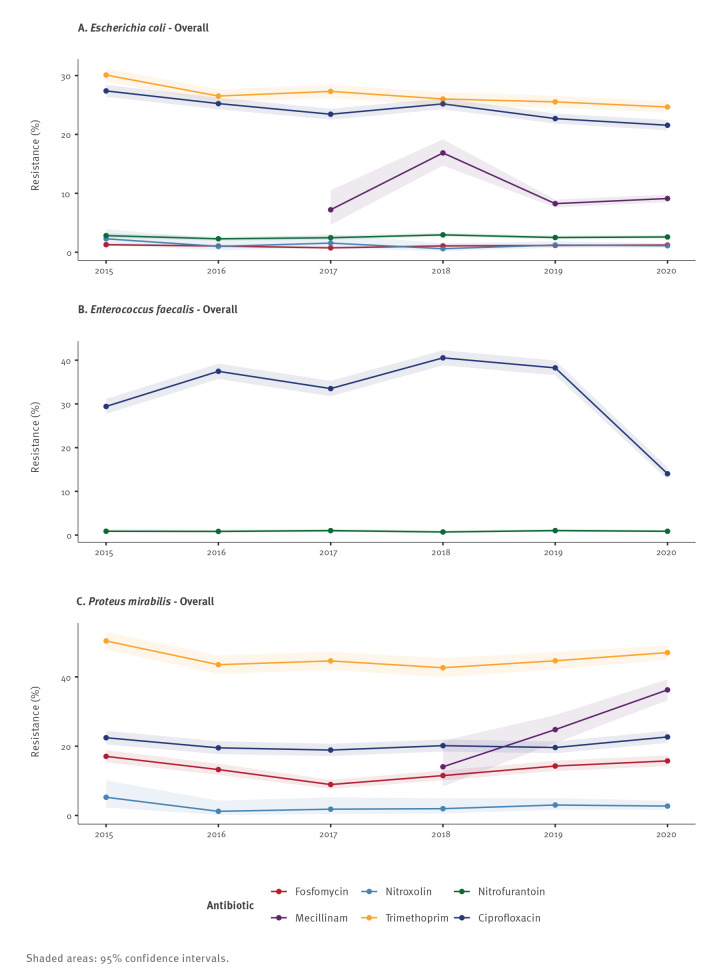
Antimicrobial resistance of bacteria detected in midstream specimens of urine of male outpatients, Germany, 2015–2020 (n = 84,377)

Figure 1 illustrates the development in unstratified data of AMR against first-line therapeutics including TMP and CIP over the years 2015 to 2020. The *E. coli* isolates showed low and stable resistance rates below 3% against fosfomycin, nitroxoline and nitrofurantoin over time (Figure 1A). In contrast, resistance of *E. coli* against mecillinam fluctuated over time to 9.1% (95% CI: 8.5–9.7) in 2020 (Figure 1A). Resistance rates of *E. coli* against CIP and TMP were high but have decreased statistically significantly since 2015 (Figure 1A). In 2020, the prevalence of *E. coli* isolates resistant to CIP and TMP was 21.6% (95% CI: 20.7–22.5) and 24.7% (95% CI: 23.7–25.6), respectively (Supplementary Table S3 lists the AMR rates in *E. coli* over time).

Shaded areas: 95% confidence intervals.

**Figure 2:**
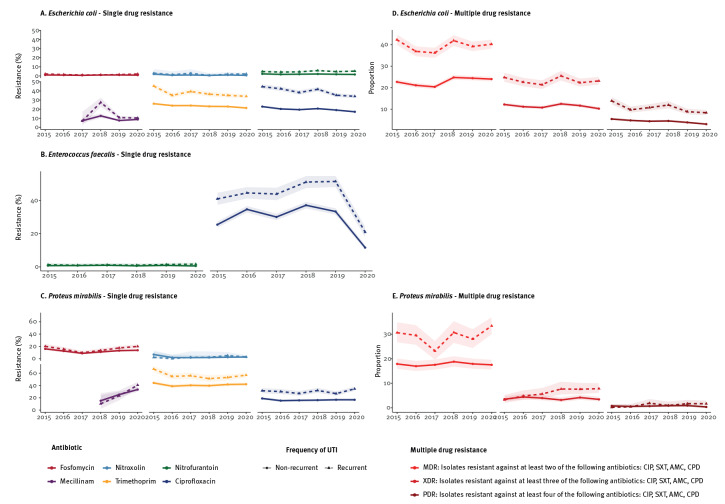
Single and multiple antimicrobial resistance of bacteria detected in midstream specimens of urine of male outpatients stratified by recurrence, Germany, 2015–2020 (n = 84,377)

Figure 2 illustrates the development of AMR and MDR stratified by recurrence. Except for fosfomycin and nitroxoline, resistance among *E. coli* isolates derived from recurrent UTI was statistically significantly higher than among *E.coli* isolates derived from non-recurrent UTI (Figure 2A). Especially for TMP and CIP, these differences appeared to be highly relevant. In 2020, 21.3% (95% CI: 20.2–22.4) of tested *E. coli* isolates received from non-recurrent were resistant against TMP compared with 34.1% (95% CI: 32.1–36.2) for recurrent UTI (Figure 2A; Supplementary Table S3). The MDR, XDR and PDR among *E. coli* differed statistically significantly between recurrent and non-recurrent UTI. The PDR declined over time (Figure 2D; Supplementary Table S3).

*Enterococcus faecalis* was susceptible against nitrofurantoin (Figure 1B). This pattern did not change by stratification (Figure 2B). In contrast, *En. faecalis* isolates tested against CIP showed high resistance. The variation in CIP resistance ranged from a maximum of 37.2% (95% CI: 35.2–39.1) in 2018 to a minimum of 11.6% (95% CI: 10.2–13.2) in 2020 (Figure 1B); detailed AMR rates in *En. faecalis* over time are listed in Supplementary Table S4.

Figure 1C illustrates resistance rates of *P. mirabilis* isolates over time. Mecillinam resistance increased constantly from 2018 to a maximum of 36.3% (95% CI: 33.3–39.3) in 2020 (Figure 1C); for the detailed AMR rates in *P. mirabilis* over time see Supplementary Table S5. Resistance of *P. mirabilis* against TMP remained unchanged, with resistance rates of 47.0% (95% CI: 44.9–49.2) in 2020. The resistance of *P. mirabilis* against CIP increased after an initial decrease statistically significantly to a maximum of 22.7% (95% CI: 21.0–24.5) in 2020. Stratification revealed statistically significantly higher resistance rates against TMP and CIP among *P. mirabilis* isolates recovered from patients with recurrent UTI (Figure 2C). There was a statistically significant difference in MDR between *P. mirabilis* isolates derived from recurrent vs non-recurrent UTI (Figure 2E; Supplementary Table S3).

Table 5 represents results of multivariable logistic regression models performed to identify risk factors for resistance of *E. coli* against TMP and CIP. Resistance against TMP had an independent positive association with the age group ≥ 90 years and with recurrent UTI and an independent negative association with laboratory sites in the regions East and South (Table 5). Resistance to CIP was independently positively associated with the age groups 60–89 and ≥ 90 years and with recurrent UTI and was independently negatively associated with laboratory sites in the regions East and South (Table 5).

**Table 5:** Multivariable logistic regression models obtaining factors associated with resistance of *Escherichia coli* isolates against trimethoprim and ciprofloxacin, male urinary tract infections, Germany, 2015–2020 (n = 49,652)

Variable/category	Trimethoprim			Ciprofloxacin		
	OR	95% CI	p value	OR	95% CI	p value
**Age (years)**						
18–29	Reference			Reference		
30–59	0.86	0.73–1.01	0.06	0.95	0.81–1.11	0.5
60–89	0.99	0.85–1.16	0.9	1.32	1.13–1.55	< 0.001
≥ 90	1.48	1.2–1.82	< 0.001	1.65	1.34–2.03	< 0.001
**Frequency of UTI last 6 months**						
Non-recurrent UTI	Reference			Reference		
Recurrent^a^ UTI	1.92	1.82–2.02	< 0.001	2.53	2.42–2.65	< 0.001
**Region**						
North	Reference			Reference		
East	0.72	0.61–0.85	< 0.001	0.82	0.76–0.88	< 0.001
South	0.91	0.85–0.97	< 0.001	0.84	0.78–0.9	< 0.001
West	1.04	0.98–1.09	0.18	1	0.95–1.05	0.99

CI: confidence interval; OR: odds ratio; UTI: urinary tract infection.

^a^ More than one positive urine culture in the last 6 month.

## Discussion

This study evaluated midstream urine specimens collected from a large cohort of male outpatients between the years 2015 and 2020 in Germany. The three most frequent bacteria *E. coli*, *En. faecalis* and *P. mirabilis*, accounted for 38.4%, 16.5% and 9.3% of all isolates, respectively. This distribution indicates distinct heterogeneity in the bacteria causing UTI in men when compared with women where *E. coli* causes most UTI. Resistance rates of all three bacteria against TMP and CIP were in total above 20%. Owing to a lack of data and the high resistance against TMP and CIP, there may be a need to further adapt treatment recommendations for men with UTI in outpatient settings.

The frequency of *E. coli* causing UTI in women is around 70% [23,24] compared with a frequency of 38.4% in men according to our findings. This is in line with previous observations that *E. coli* cause a significantly smaller proportion of UTI in men than in women [25]. In contrast, *En. faecalis* and *P. mirabilis* are more prevalent in men than in women [23,24].

In-depth analyses of AMR of *E. coli* revealed resistance rates below 3% against fosfomycin, nitroxoline and nitrofurantoin in our study. The same has been shown in studies with female outpatients [21,24]. Importantly, the resistance rates against these antibiotics did not increase over time, nor did they differ when stratified by recurrence. Resistance rates of *En. faecalis* against nitrofurantoin remained stable over time at around 1%. Similar rates have been reported for *En. faecalis* by the national antibiotic resistance surveillance of the Robert Koch Institute [26]. *Proteus mirabilis* isolates had low and constant resistance rates of 3% against nitroxoline. Resistance rates against fosfomycin were higher with 14% and increased over time. These results indicate a potential for the use of fosfomycin, nitroxoline and nitrofurantoin in men. Especially the high activity of nitrofurantoin before culture result (75%) makes nitrofurantoin a suitable empirical therapeutic, which is reflected in other studies [27]. Nevertheless, it is important to be aware of the poor tissue penetration of this antibiotic despite its high activity in vitro [28]. Considering our findings as well as reported case reports, fosfomycin could also be a promising therapeutic option for male outpatients, although its activity against enterococci is unclear [14]. Of note, important issues such as initial dosing, duration of intake and prescribing intervals for fosfomycin in outpatient treatment of complicated UTI are not yet fully resolved. Furthermore, as stated in other studies, we could verify that *En. faecalis* occurred more frequently in polymicrobial (22.9%) rather than monomicrobial infections (12%) [29]. Nevertheless, the importance of treating enterococci in mixed infections has not yet been clarified [30].

The resistance rates of *E. coli* against mecillinam were higher, around 9%, although earlier studies in female patients saw lower rates than that [21,24]. Resistance of *P. mirabilis* against mecillinam was high (31%) and increased over time. However, this consistent increase remains unexplained and therefore needs to be interpreted with caution.

Resistance rates of *E. coli and P. mirabilis* isolates against TMP were notably high with 27% and 46%. *Escherichia coli, En. faecalis* and *P. mirabilis* isolates had high resistance rates against CIP with respectively 24%, 33% and 21%. The resistance of *En. faecalis* against CIP declined strongly in 2020. This decline, however, remains unexplained and needs to be interpreted with caution. While there were no changes in EUCAST breakpoints for susceptibility of enterococci against fluoroquinolones from 2019 to 2020, some of the laboratories changed from direct susceptibility testing to norfloxacin screen. Although accompanying factors such as the coronavirus disease (COVID 19) pandemic could be an underlying reason for this development, there is no explanation why other antibiotic/pathogen combinations were not similarly affected. Irrespective of the pathogen, the level of resistance against CIP and TMP differed statistically significantly when stratified by recurrence. It is likely that this effect reflects the role of CIP and TMP as preferred treatment options. It is therefore important to prevent further selection of TMP- or CIP-resistant bacteria. Their use as both empirical and calculated therapeutics for UTI in men in outpatient settings should be re-evaluated in future studies, possibly leading to recommendations contrary to existing national and international guidelines [4]. Furthermore, these results support findings that, irrespective of sex, the empirical use of fluoroquinolones for treatment of UTI is not an inappropriate therapy in cases where UTI are likely to be caused by *E. coli* [31].

Resistance of *E. coli* against TMP and CIP was positively associated with recurrent UTI and age  ≥ 90 years as well as negatively associated with laboratory sites in the regions East and South. Recurrent UTI and high age have already been observed as independent risk factors for AMR in female patients with UTI [23,32]. Regional differences, however, need to be interpreted with caution. Although there is evidence that antimicrobial susceptibility in other pathogens differs between regions [33], our data do not provide information on the patients’ health status or type of healthcare providers. Therefore, we cannot exclude bias due to structural regional differences. In addition, laboratory sites do necessarily reflect the geographical locations of the practitioner sending the samples, even though they commonly receive most specimens from a closer vicinity. 

In this study, we included routinely collected midstream urine specimens from male outpatients and included only those bacterial isolates that are known to cause UTI. We thereby tried to include only patients with UTI, while the diagnosis UTI was not made clinically. Urine specimens could be sent to laboratories for other reasons which would result in biased estimates. But considering that German guidelines recommend pre-treatment urine culture for men with suspected UTI, urine cultures with positive and relevant results are likely to represent the clinical diagnosis UTI in men [4]. That being said, adherence to this guideline is not well known. Nevertheless, because male UTI is predominantly interpretated as complicated UTI [7] and because pre-treatment urine cultures are recommended for complicated UTI irrespective of sex, the diagnostic procedure is intuitive and therefore adherence to the guideline is likely. Nevertheless, it is important to note that non-adherence (e.g. in rare cases of uncomplicated UTI in men) is likely to bias the estimates towards higher resistance rates.

The retrospective design of this study inherently gives risk for bias. For example, the missing differentiation between uncomplicated and complicated UTI could bias the estimates towards lower resistance rates among uncomplicated UTI, as shown for female patients [23]. Further we did not have information on underlying clinical conditions or prior antimicrobial treatment. Both aspects are needed for reliable estimates. To reduce bias due to previous antimicrobial treatment, we stratified the data by recurrence of UTI. Nevertheless, antibiotics are prescribed for multiple clinical concerns other than UTI and therefore we cannot exclude that our patients had used antimicrobial drugs before their UTI. Moreover, information on underlying clinical conditions as well as previous use of antimicrobial drugs could be used to better identify risk factors for AMR and would present an opportunity to derive highly reliable therapy recommendations. To achieve such recommendations, a study conducted with a multicentre and prospective design would be necessary to fill the existing evidence gap in male UTI.

## Conclusion

The distribution of the detected bacteria indicates high heterogeneity of UTI in men and supports the current guideline recommendation to always perform urine culture before therapy when suspecting UTI in men. The high resistance against TMP and CIP, especially in recurrent UTI, is of concern. As the resistance of tested isolates against fosfomycin and nitrofurantoin was relatively low, we conclude that these antibiotics should be considered as primary therapy options and the use of CIP and TMP should be limited.

## Supplementary Data

438000 Supplement
