# Erratum for Euro Surveill. 2021;27(30)

**DOI:** 10.2807/1560-7917.ES.2022.27.31.220804e

**Published:** 2022-08-04

**Authors:** Eurosurveillance editorial team

**Affiliations:** 1 European Centre for Disease Prevention and Control (ECDC), Stockholm, Sweden

In the article ‘High antimicrobial resistance in urinary tract infections in male outpatients in routine laboratory data, Germany, 2015 to 2020’ by Salm et al. published on 28 July 2022, footnote c in Table 3 was misassigned in the originally published version. This was corrected on 1 August 2022. The remaining footnotes in this table were reordered in the context of this change. We apologise for the mistake.

