# Supplementary material for: High antimicrobial resistance in urinary tract infections in male outpatients in routine laboratory data, Germany, 2015 to 2020
Source: Euro Surveill. 2022 Jul 28;27(30):2101012. doi: 10.2807/1560-7917.ES.2022.27.30.2101012 (PMC9336165; doi:10.2807/1560-7917.ES.2022.27.30.2101012)
Supplement: Supplement [file 21-01012_SALM_Supplement.pdf]

This supplementary material is hosted by *Eurosurveillance* as supporting information alongside the article *High antimicrobial resistance in urinary tract infections in male outpatients: frequency and antimicrobial resistance in urinary tract infections in male outpatients in routine laboratory data, Germany, 2015 to 2020* on behalf of the authors who remain responsible for the accuracy and appropriateness of the content. The same standards for ethics, copyright, attributions and permissions as for the article apply. Supplements are not edited by Eurosurveillance and the journal is not responsible for the maintenance of any links or email addresses provided therein.

S1: Laboratory sites of participating laboratories

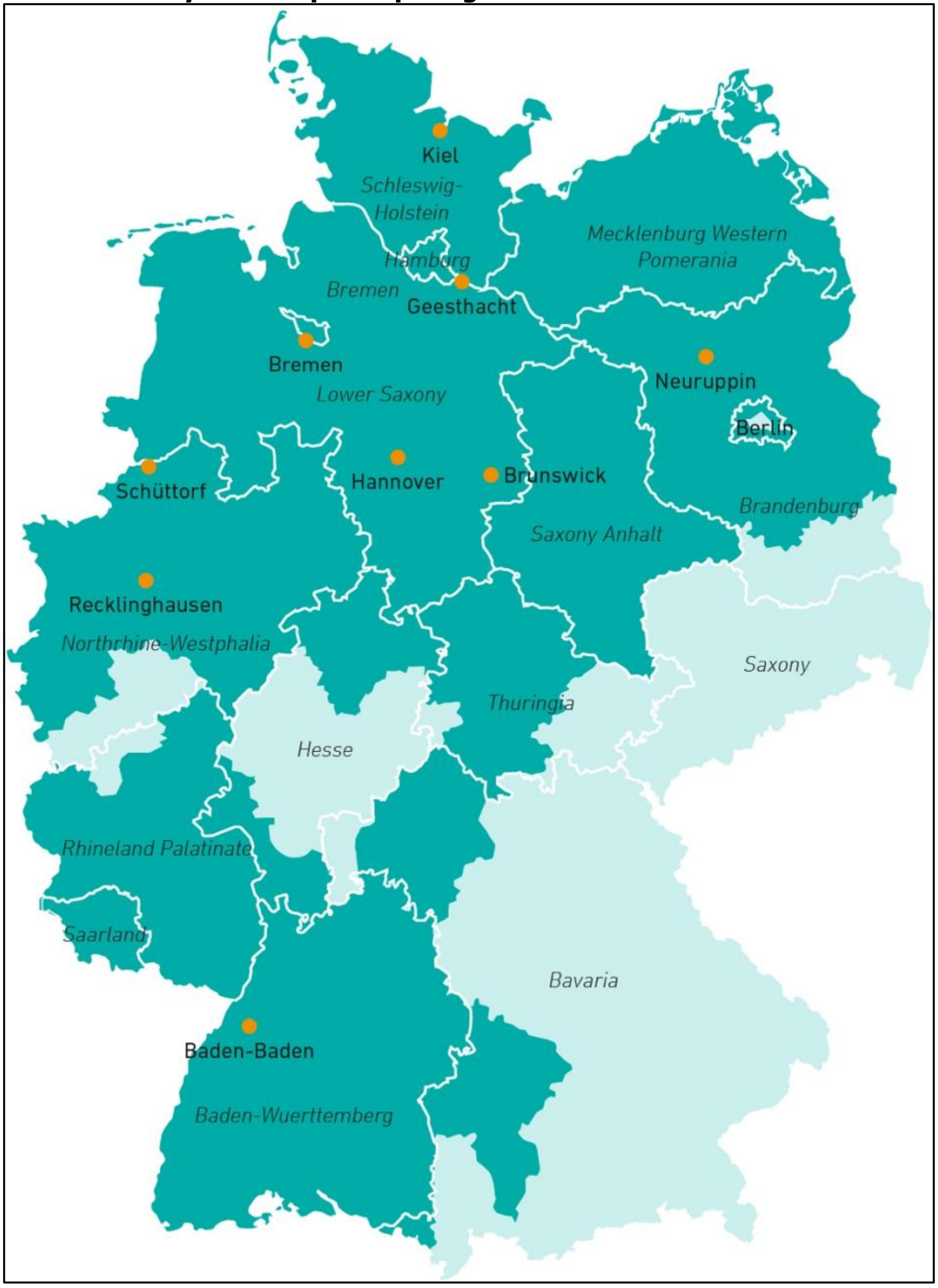

Locations of the laboratories and the areas of outpatient services that send urine samples of male adult patients between 2015 and 2020. Laboratories are marked in orange

S2: Data processing steps

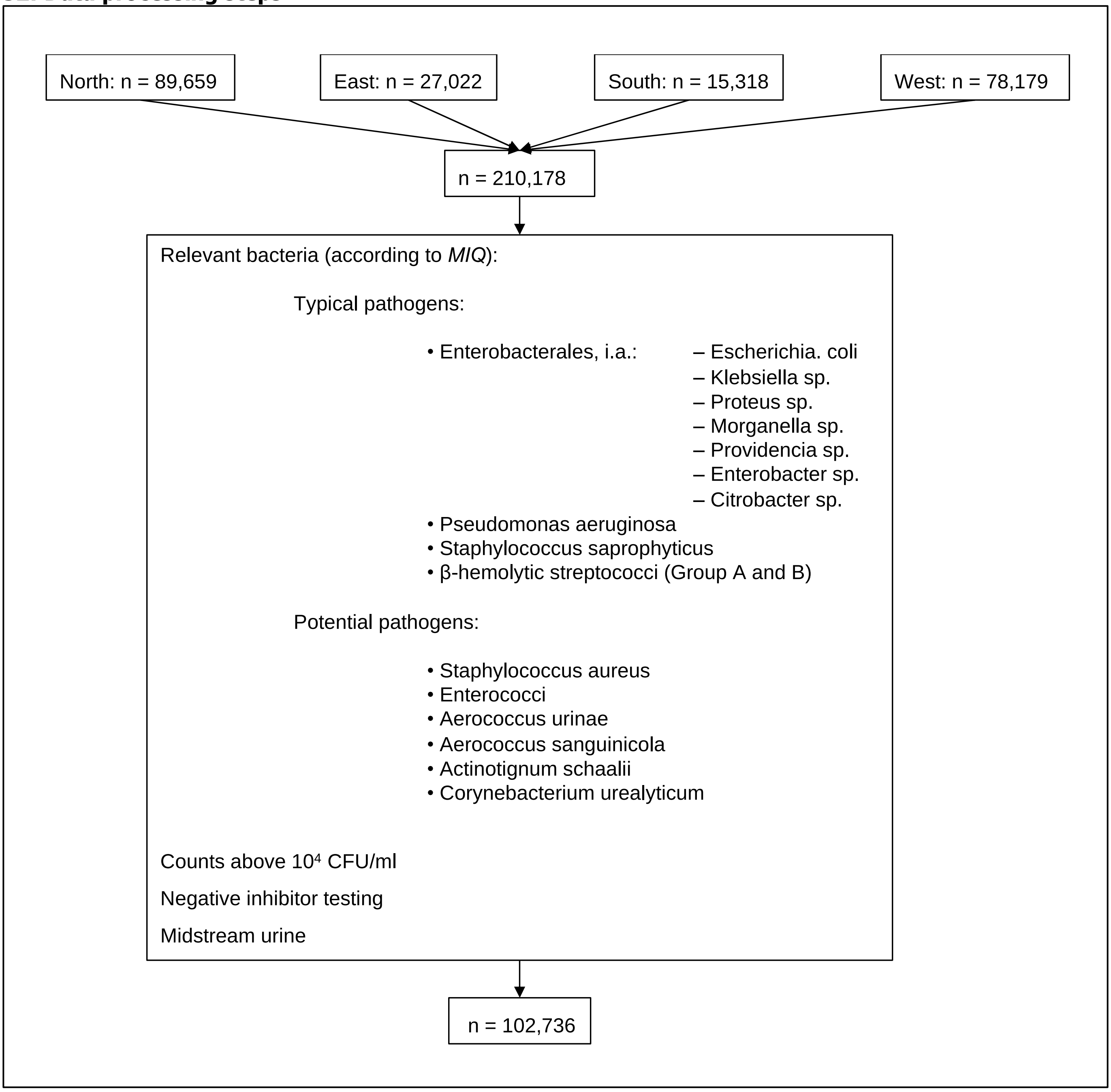

S3: Antimicrobial resistance rates in *E. coli* per year from 2015 to 2020

Year	R in %	95% CI	n tested	Antibiotic	Frequency
2015	1.28	1.04-1.56	7,594	Fosfomycin	Total
2016	1.05	0.84-1.31	7,587	Fosfomycin	Total
2017	0.74	0.57-0.96	8,063	Fosfomycin	Total
2018	1.07	0.87-1.32	8,561	Fosfomycin	Total
2019	1.15	0.94-1.39	9,387	Fosfomycin	Total
2020	1.22	1-1.48	8,354	Fosfomycin	Total
2015	2.28	1.18-3.94	527	Nitroxolin	Total
2016	0.99	0.32-2.3	503	Nitroxolin	Total
2017	1.55	0.67-3.03	517	Nitroxolin	Total
2018	0.60	0.16-1.54	663	Nitroxolin	Total
2019	1.22	0.75-1.88	1,641	Nitroxolin	Total
2020	1.10	0.76-1.53	3,102	Nitroxolin	Total
2015	2.82	2.45-3.22	7,347	Nitrofurantoin	Total
2016	2.28	1.95-2.64	7,548	Nitrofurantoin	Total
2017	2.46	2.13-2.83	7,882	Nitrofurantoin	Total
2018	2.95	2.6-3.33	8,570	Nitrofurantoin	Total
2019	2.50	2.19-2.83	9,373	Nitrofurantoin	Total
2020	2.59	2.26-2.96	8,372	Nitrofurantoin	Total
2017	7.21	4.67-10.53	333	Mecillinam	Total
2018	16.86	14.72-19.17	1,133	Mecillinam	Total
2019	8.25	7.6-8.93	6,693	Mecillinam	Total
2020	9.12	8.5-9.78	7,882	Mecillinam	Total
2015	30.08	28.96-31.21	6,487	Trimethoprim	Total
2016	26.52	25.4-27.65	6,019	Trimethoprim	Total
2017	27.31	26.18-28.46	5,962	Trimethoprim	Total
2018	26.02	24.86-27.21	5,465	Trimethoprim	Total
2019	25.53	24.52-26.56	7,098	Trimethoprim	Total
2020	24.66	23.71-25.62	7,945	Trimethoprim	Total
2015	27.39	26.39-28.41	7,612	Ciprofloxacin	Total
2016	25.24	24.27-26.23	7,595	Ciprofloxacin	Total
2017	23.43	22.51-24.37	8,083	Ciprofloxacin	Total
2018	25.19	24.27-26.12	8,599	Ciprofloxacin	Total
2019	22.69	21.85-23.55	9,400	Ciprofloxacin	Total
2020	21.56	20.69-22.46	8,389	Ciprofloxacin	Total
2015	1.03	0.79-1.32	6,004	Fosfomycin	Non-recurrent
2016	0.92	0.69-1.2	5,874	Fosfomycin	Non-recurrent
2017	0.66	0.48-0.89	6,355	Fosfomycin	Non-recurrent
2018	1.03	0.8-1.3	6,716	Fosfomycin	Non-recurrent
2019	1.05	0.83-1.31	7,256	Fosfomycin	Non-recurrent
2020	0.89	0.67-1.16	6,160	Fosfomycin	Non-recurrent
2015	2.20	1.54-3.05	1,590	Fosfomycin	Recurrent
2016	1.52	0.99-2.22	1,713	Fosfomycin	Recurrent
2017	1.05	0.63-1.66	1,708	Fosfomycin	Recurrent
2018	1.25	0.79-1.86	1,845	Fosfomycin	Recurrent
2019	1.50	1.03-2.11	2,131	Fosfomycin	Recurrent
2020	2.14	1.58-2.84	2,194	Fosfomycin	Recurrent
2015	2.03	0.88-3.95	395	Nitroxolin	Non-recurrent
2016	0.82	0.17-2.38	365	Nitroxolin	Non-recurrent
2017	1.06	0.29-2.68	379	Nitroxolin	Non-recurrent
2018	0.61	0.13-1.77	493	Nitroxolin	Non-recurrent
2019	1.05	0.58-1.76	1,329	Nitroxolin	Non-recurrent
2020	0.63	0.35-1.06	2,210	Nitroxolin	Non-recurrent

S3: Antimicrobial resistance rates in *E. coli* per year from 2015 to 2020

Year	R in %	95% CI	n tested	Antibiotic	Frequency
2015	3.03	0.83-7.58	132	Nitroxolin	Recurrent
2016	1.45	0.18-5.14	138	Nitroxolin	Recurrent
2017	2.90	0.8-7.26	138	Nitroxolin	Recurrent
2018	0.59	0.01-3.23	170	Nitroxolin	Recurrent
2019	1.92	0.71-4.14	312	Nitroxolin	Recurrent
2020	2.24	1.37-3.44	892	Nitroxolin	Recurrent
2015	2.28	1.91-2.7	5,795	Nitrofurantoin	Non-recurrent
2016	1.71	1.39-2.08	5,847	Nitrofurantoin	Non-recurrent
2017	1.91	1.59-2.29	6,217	Nitrofurantoin	Non-recurrent
2018	2.14	1.81-2.52	6,719	Nitrofurantoin	Non-recurrent
2019	1.84	1.54-2.17	7,244	Nitrofurantoin	Non-recurrent
2020	1.64	1.33-1.98	6,173	Nitrofurantoin	Non-recurrent
2015	4.83	3.82-6.02	1,552	Nitrofurantoin	Recurrent
2016	4.23	3.33-5.3	1,701	Nitrofurantoin	Recurrent
2017	4.50	3.56-5.61	1,665	Nitrofurantoin	Recurrent
2018	5.89	4.86-7.06	1,851	Nitrofurantoin	Recurrent
2019	4.74	3.88-5.73	2,129	Nitrofurantoin	Recurrent
2020	5.28	4.38-6.29	2,199	Nitrofurantoin	Recurrent
2017	7.25	4.48-10.97	276	Mecillinam	Non-recurrent
2018	12.62	10.41-15.11	808	Mecillinam	Non-recurrent
2019	7.54	6.84-8.29	5,211	Mecillinam	Non-recurrent
2020	8.69	7.98-9.45	5,810	Mecillinam	Non-recurrent
2017	7.02	1.95-17	57	Mecillinam	Recurrent
2018	27.38	22.61-32.58	325	Mecillinam	Recurrent
2019	10.73	9.2-12.42	1,482	Mecillinam	Recurrent
2020	10.33	9.05-11.72	2,072	Mecillinam	Recurrent
2015	26.00	24.8-27.22	5,123	Trimethoprim	Non-recurrent
2016	23.88	22.65-25.14	4,611	Trimethoprim	Non-recurrent
2017	23.97	22.75-25.22	4,669	Trimethoprim	Non-recurrent
2018	23.06	21.8-24.35	4,259	Trimethoprim	Non-recurrent
2019	22.88	21.78-24.01	5,568	Trimethoprim	Non-recurrent
2020	21.29	20.24-22.36	5,858	Trimethoprim	Non-recurrent
2015	45.38	42.71-48.07	1,364	Trimethoprim	Recurrent
2016	35.16	32.66-37.71	1,408	Trimethoprim	Recurrent
2017	39.37	36.69-42.09	1,293	Trimethoprim	Recurrent
2018	36.48	33.76-39.27	1,206	Trimethoprim	Recurrent
2019	35.16	32.77-37.62	1,530	Trimethoprim	Recurrent
2020	34.12	32.08-36.2	2,087	Trimethoprim	Recurrent
2015	22.81	21.76-23.89	6,019	Ciprofloxacin	Non-recurrent
2016	20.25	19.23-21.3	5,882	Ciprofloxacin	Non-recurrent
2017	19.45	18.48-20.44	6,371	Ciprofloxacin	Non-recurrent
2018	20.61	19.65-21.6	6,744	Ciprofloxacin	Non-recurrent
2019	18.99	18.09-19.91	7,267	Ciprofloxacin	Non-recurrent
2020	17.10	16.17-18.06	6,187	Ciprofloxacin	Non-recurrent
2015	44.70	42.23-47.18	1,593	Ciprofloxacin	Recurrent
2016	42.38	40.03-44.76	1,713	Ciprofloxacin	Recurrent
2017	38.26	35.95-40.61	1,712	Ciprofloxacin	Recurrent
2018	41.83	39.58-44.12	1,855	Ciprofloxacin	Recurrent
2019	35.30	33.27-37.37	2,133	Ciprofloxacin	Recurrent
2020	34.11	32.12-36.13	2,202	Ciprofloxacin	Recurrent

S4: Antimicrobial resistance rates in *Enterococcus faecalis* per year from 2015 to 2020

Year	R in %	95% CI	n tested	Antibiotic	Frequency
2015	0.88	0.57-1.31	2,725	Nitrofurantoin	Total
2016	0.84	0.54-1.25	2,846	Nitrofurantoin	Total
2017	1.02	0.67-1.48	2,646	Nitrofurantoin	Total
2018	0.71	0.45-1.07	3,225	Nitrofurantoin	Total
2019	1.03	0.74-1.41	3,772	Nitrofurantoin	Total
2020	0.87	0.59-1.24	3,449	Nitrofurantoin	Total
2015	29.42	27.74-31.13	2,842	Ciprofloxacin	Total
2016	37.45	35.7-39.21	2,983	Ciprofloxacin	Total
2017	33.50	31.75-35.28	2,809	Ciprofloxacin	Total
2018	40.53	38.8-42.27	3,131	Ciprofloxacin	Total
2019	38.24	36.56-39.94	3,227	Ciprofloxacin	Total
2020	14.03	12.7-15.46	2,501	Ciprofloxacin	Total
2015	0.74	0.42-1.22	2,021	Nitrofurantoin	Non-recurrent
2016	0.78	0.45-1.26	2,049	Nitrofurantoin	Non-recurrent
2017	0.95	0.58-1.49	1,990	Nitrofurantoin	Non-recurrent
2018	0.61	0.34-1.01	2,447	Nitrofurantoin	Non-recurrent
2019	0.89	0.58-1.32	2,795	Nitrofurantoin	Non-recurrent
2020	0.54	0.29-0.92	2,413	Nitrofurantoin	Non-recurrent
2015	1.28	0.59-2.41	704	Nitrofurantoin	Recurrent
2016	1.00	0.43-1.97	797	Nitrofurantoin	Recurrent
2017	1.22	0.53-2.39	656	Nitrofurantoin	Recurrent
2018	1.03	0.44-2.02	778	Nitrofurantoin	Recurrent
2019	1.43	0.79-2.39	977	Nitrofurantoin	Recurrent
2020	1.64	0.96-2.61	1,036	Nitrofurantoin	Recurrent
2015	25.46	23.62-27.37	2,117	Ciprofloxacin	Non-recurrent
2016	34.68	32.67-36.74	2,151	Ciprofloxacin	Non-recurrent
2017	30.07	28.12-32.07	2,112	Ciprofloxacin	Non-recurrent
2018	37.15	35.2-39.13	2,374	Ciprofloxacin	Non-recurrent
2019	33.36	31.46-35.31	2,359	Ciprofloxacin	Non-recurrent
2020	11.63	10.21-13.18	1,857	Ciprofloxacin	Non-recurrent
2015	40.97	37.36-44.64	725	Ciprofloxacin	Recurrent
2016	44.59	41.18-48.04	832	Ciprofloxacin	Recurrent
2017	43.90	40.18-47.68	697	Ciprofloxacin	Recurrent
2018	51.12	47.5-54.74	757	Ciprofloxacin	Recurrent
2019	51.50	48.12-54.87	868	Ciprofloxacin	Recurrent
2020	20.96	17.88-24.31	644	Ciprofloxacin	Recurrent

S5: Antimicrobial resistance of *Proteus mirabilis* per year from 2015 to 2020

Year	R in %	95% CI	n tested	Antibiotic	Frequency
2015	17.06	15.34-18.89	1,776	Fosfomycin	Total
2016	13.25	11.7-14.93	1,758	Fosfomycin	Total
2017	8.93	7.69-10.28	1,938	Fosfomycin	Total
2018	11.51	10.16-12.97	2,050	Fosfomycin	Total
2019	14.27	12.81-15.82	2,152	Fosfomycin	Total
2020	15.75	14.26-17.33	2,235	Fosfomycin	Total
2015	5.26	2.3-10.11	152	Nitroxolin	Total
2016	1.20	0.15-4.28	166	Nitroxolin	Total
2017	1.82	0.38-5.22	165	Nitroxolin	Total
2018	1.96	0.54-4.94	204	Nitroxolin	Total
2019	3.02	1.66-5.01	464	Nitroxolin	Total
2020	2.71	1.61-4.25	664	Nitroxolin	Total
2018	14.05	8.4-21.54	121	Mecillinam	Total
2019	24.78	20.92-28.97	464	Mecillinam	Total
2020	36.25	33.27-39.3	1,007	Mecillinam	Total
2015	50.37	47.81-52.93	1,501	Trimethoprim	Total
2016	43.52	40.87-46.19	1,365	Trimethoprim	Total
2017	44.61	41.96-47.29	1,374	Trimethoprim	Total
2018	42.64	39.87-45.44	1,243	Trimethoprim	Total
2019	44.65	42.15-47.18	1,534	Trimethoprim	Total
2020	47.01	44.88-49.15	2,140	Trimethoprim	Total
2015	22.44	20.52-24.45	1,778	Ciprofloxacin	Total
2016	19.52	17.7-21.45	1,762	Ciprofloxacin	Total
2017	18.88	17.16-20.69	1,939	Ciprofloxacin	Total
2018	20.15	18.45-21.95	2,069	Ciprofloxacin	Total
2019	19.61	17.95-21.35	2,157	Ciprofloxacin	Total
2020	22.66	20.95-24.45	2,259	Ciprofloxacin	Total
2015	44.23	41.26-47.23	1,092	Trimethoprim	Non-recurrent
2016	38.91	35.82-42.06	969	Trimethoprim	Non-recurrent
2017	40.32	37.26-43.44	997	Trimethoprim	Non-recurrent
2018	39.74	36.58-42.97	931	Trimethoprim	Non-recurrent
2019	41.70	38.82-44.63	1,139	Trimethoprim	Non-recurrent
2020	42.11	39.54-44.72	1,432	Trimethoprim	Non-recurrent
2015	66.75	61.95-71.3	409	Trimethoprim	Recurrent
2016	54.80	49.75-59.77	396	Trimethoprim	Recurrent
2017	55.97	50.79-61.05	377	Trimethoprim	Recurrent
2018	51.28	45.59-56.95	312	Trimethoprim	Recurrent
2019	53.16	48.11-58.17	395	Trimethoprim	Recurrent
2020	56.92	53.18-60.6	708	Trimethoprim	Recurrent
2015	15.91	13.95-18.03	1,282	Fosfomycin	Non-recurrent
2016	12.38	10.61-14.33	1,260	Fosfomycin	Non-recurrent
2017	8.63	7.22-10.22	1,413	Fosfomycin	Non-recurrent
2018	10.88	9.37-12.55	1,535	Fosfomycin	Non-recurrent
2019	13.04	11.4-14.82	1,549	Fosfomycin	Non-recurrent
2020	13.66	11.96-15.51	1,493	Fosfomycin	Non-recurrent
2015	20.04	16.6-23.85	494	Fosfomycin	Recurrent
2016	15.46	12.4-18.94	498	Fosfomycin	Recurrent
2017	9.71	7.32-12.57	525	Fosfomycin	Recurrent
2018	13.40	10.58-16.65	515	Fosfomycin	Recurrent
2019	17.41	14.47-20.68	603	Fosfomycin	Recurrent
2020	19.95	17.13-23.01	742	Fosfomycin	Recurrent
2015	6.73	2.75-13.38	104	Nitroxolin	Non-recurrent

S5: Antimicrobial resistance of *Proteus mirabilis* per year from 2015 to 2020

Year	R in %	95% CI	n tested	Antibiotic	Frequency
2016	1.83	0.22-6.47	109	Nitroxolin	Non-recurrent
2017	1.65	0.2-5.84	121	Nitroxolin	Non-recurrent
2018	1.89	0.39-5.41	159	Nitroxolin	Non-recurrent
2019	2.31	1-4.5	346	Nitroxolin	Non-recurrent
2020	2.61	1.31-4.63	421	Nitroxolin	Non-recurrent
2015	2.08	0.05-11.07	48	Nitroxolin	Recurrent
2016	0.00	0-6.27	57	Nitroxolin	Recurrent
2017	2.27	0.06-12.02	44	Nitroxolin	Recurrent
2018	2.22	0.06-11.77	45	Nitroxolin	Recurrent
2019	5.08	1.89-10.74	118	Nitroxolin	Recurrent
2020	2.88	1.17-5.84	243	Nitroxolin	Recurrent
2018	15.22	8.58-24.21	92	Mecillinam	Non-recurrent
2019	25.29	20.8-30.2	348	Mecillinam	Non-recurrent
2020	33.44	29.73-37.31	619	Mecillinam	Non-recurrent
2018	10.34	2.19-27.35	29	Mecillinam	Recurrent
2019	23.28	15.93-32.03	116	Mecillinam	Recurrent
2020	40.72	35.79-45.79	388	Mecillinam	Recurrent
2015	18.94	16.83-21.19	1,283	Ciprofloxacin	Non-recurrent
2016	15.35	13.4-17.46	1,264	Ciprofloxacin	Non-recurrent
2017	15.84	13.98-17.85	1,414	Ciprofloxacin	Non-recurrent
2018	16.19	14.39-18.12	1,550	Ciprofloxacin	Non-recurrent
2019	16.81	14.98-18.76	1,553	Ciprofloxacin	Non-recurrent
2020	16.79	14.94-18.77	1,513	Ciprofloxacin	Non-recurrent
2015	31.52	27.44-35.81	495	Ciprofloxacin	Recurrent
2016	30.12	26.12-34.36	498	Ciprofloxacin	Recurrent
2017	27.05	23.29-31.06	525	Ciprofloxacin	Recurrent
2018	31.98	27.99-36.19	519	Ciprofloxacin	Recurrent
2019	26.82	23.33-30.54	604	Ciprofloxacin	Recurrent
2020	34.58	31.17-38.12	746	Ciprofloxacin	Recurrent

S6: Multiple drug resistance in *Escherichia coli* per year from 2015 to 2020

Year	Proportion	95% CI	n tested	Multiple resistance	Stratified	Pathogen
2015	26.79	25.81-27.8	7591	MDR	Total	E. coli
2016	24.68	23.73-25.67	7580	MDR	Total	E. coli
2017	23.77	22.83-24.74	7605	MDR	Total	E. coli
2018	28.28	27.25-29.33	7239	MDR	Total	E. coli
2019	27.76	26.8-28.73	8268	MDR	Total	E. coli
2020	28.26	27.28-29.25	8030	MDR	Total	E. coli
2015	22.70	21.65-23.77	6001	MDR	Non-recurrent UTI	E. coli
2016	21.13	20.11-22.2	5872	MDR	Non-recurrent UTI	E. coli
2017	20.41	19.41-21.45	5983	MDR	Non-recurrent UTI	E. coli
2018	24.77	23.68-25.91	5756	MDR	Non-recurrent UTI	E. coli
2019	24.43	23.4-25.5	6401	MDR	Non-recurrent UTI	E. coli
2020	24.01	22.94-25.11	5923	MDR	Non-recurrent UTI	E. coli
2015	42.26	39.86-44.71	1590	MDR	Recurrent UTI	E. coli
2016	36.89	34.63-39.2	1708	MDR	Recurrent UTI	E. coli
2017	36.19	33.88-38.56	1622	MDR	Recurrent UTI	E. coli
2018	41.87	39.39-44.41	1483	MDR	Recurrent UTI	E. coli
2019	39.15	36.96-41.39	1867	MDR	Recurrent UTI	E. coli
2020	40.20	38.12-42.31	2107	MDR	Recurrent UTI	E. coli
2015	14.86	14.08-15.68	7591	XDR	Total	E. coli
2016	13.77	13.02-14.57	7580	XDR	Total	E. coli
2017	13.06	12.32-13.83	7605	XDR	Total	E. coli
2018	15.17	14.36-16.01	7239	XDR	Total	E. coli
2019	14.10	13.37-14.87	8268	XDR	Total	E. coli
2020	13.67	12.94-14.44	8030	XDR	Total	E. coli
2015	12.23	11.43-13.08	6001	XDR	Non-recurrent UTI	E. coli
2016	11.21	10.42-12.04	5872	XDR	Non-recurrent UTI	E. coli
2017	10.80	10.04-11.61	5983	XDR	Non-recurrent UTI	E. coli
2018	12.51	11.68-13.39	5756	XDR	Non-recurrent UTI	E. coli
2019	11.70	10.94-12.51	6401	XDR	Non-recurrent UTI	E. coli
2020	10.30	9.55-11.1	5923	XDR	Non-recurrent UTI	E. coli
2015	24.78	22.72-26.96	1590	XDR	Recurrent UTI	E. coli
2016	22.60	20.68-24.65	1708	XDR	Recurrent UTI	E. coli
2017	21.39	19.46-23.46	1622	XDR	Recurrent UTI	E. coli
2018	25.49	23.33-27.77	1483	XDR	Recurrent UTI	E. coli
2019	22.34	20.5-24.28	1867	XDR	Recurrent UTI	E. coli
2020	23.16	21.41-25.01	2107	XDR	Recurrent UTI	E. coli
2015	7.25	6.68-7.85	7591	PDR	Total	E. coli
2016	5.95	5.44-6.51	7580	PDR	Total	E. coli
2017	5.80	5.3-6.35	7605	PDR	Total	E. coli
2018	6.11	5.58-6.68	7239	PDR	Total	E. coli
2019	5.03	4.58-5.52	8268	PDR	Total	E. coli
2020	4.50	4.06-4.97	8030	PDR	Total	E. coli
2015	5.48	4.93-6.09	6001	PDR	Non-recurrent UTI	E. coli
2016	4.84	4.32-5.42	5872	PDR	Non-recurrent UTI	E. coli
2017	4.45	3.95-5	5983	PDR	Non-recurrent UTI	E. coli
2018	4.59	4.08-5.16	5756	PDR	Non-recurrent UTI	E. coli
2019	3.91	3.46-4.41	6401	PDR	Non-recurrent UTI	E. coli
2020	3.11	2.69-3.58	5923	PDR	Non-recurrent UTI	E. coli
2015	13.90	12.28-15.69	1590	PDR	Recurrent UTI	E. coli
2016	9.78	8.46-11.28	1708	PDR	Recurrent UTI	E. coli
2017	10.79	9.37-12.4	1622	PDR	Recurrent UTI	E. coli
2018	12.00	10.44-13.76	1483	PDR	Recurrent UTI	E. coli

S6: Multiple drug resistance in *Escherichia coli* per year from 2015 to 2020

Year	Proportion	95% CI	n tested	Multiple resistance	Stratified	Pathogen
2019	8.89	7.68-10.27	1867	PDR	Recurrent UTI	E. coli
2020	8.40	7.29-9.66	2107	PDR	Recurrent UTI	E. coli

S7 : Multiple drug resistance in *Proteus mirabilis* per year from 2015 to 2020

Year	Proportion	95% CI	N tested	Multiple resistance	Stratified	Pathogen
2015	21.40	19.55-23.37	1771	MDR	Total	P.mirabilis
2016	20.52	18.7-22.48	1759	MDR	Total	P.mirabilis
2017	19.04	17.29-20.93	1791	MDR	Total	P.mirabilis
2018	21.72	19.82-23.74	1699	MDR	Total	P.mirabilis
2019	20.77	18.99-22.68	1863	MDR	Total	P.mirabilis
2020	22.82	21.09-24.64	2143	MDR	Total	P.mirabilis
2015	17.88	15.87-20.07	1281	MDR	Non-recurrent UTI	P.mirabilis
2016	16.97	15-19.15	1261	MDR	Non-recurrent UTI	P.mirabilis
2017	17.53	15.57-19.68	1312	MDR	Non-recurrent UTI	P.mirabilis
2018	18.80	16.75-21.03	1282	MDR	Non-recurrent UTI	P.mirabilis
2019	17.90	15.94-20.05	1335	MDR	Non-recurrent UTI	P.mirabilis
2020	17.52	15.63-19.58	1427	MDR	Non-recurrent UTI	P.mirabilis
2015	30.61	26.68-34.84	490	MDR	Recurrent UTI	P.mirabilis
2016	29.52	25.67-33.68	498	MDR	Recurrent UTI	P.mirabilis
2017	23.17	19.61-27.17	479	MDR	Recurrent UTI	P.mirabilis
2018	30.70	26.45-35.3	417	MDR	Recurrent UTI	P.mirabilis
2019	28.03	24.36-32.02	528	MDR	Recurrent UTI	P.mirabilis
2020	33.38	30.02-36.92	716	MDR	Recurrent UTI	P.mirabilis
2015	3.44	2.69-4.4	1771	XDR	Total	P.mirabilis
2016	4.55	3.67-5.63	1759	XDR	Total	P.mirabilis
2017	4.41	3.55-5.47	1791	XDR	Total	P.mirabilis
2018	4.30	3.43-5.37	1699	XDR	Total	P.mirabilis
2019	5.15	4.24-6.25	1863	XDR	Total	P.mirabilis
2020	4.90	4.06-5.9	2143	XDR	Total	P.mirabilis
2015	3.51	2.63-4.67	1281	XDR	Non-recurrent UTI	P.mirabilis
2016	4.44	3.43-5.73	1261	XDR	Non-recurrent UTI	P.mirabilis
2017	3.96	3.03-5.17	1312	XDR	Non-recurrent UTI	P.mirabilis
2018	3.20	2.36-4.32	1282	XDR	Non-recurrent UTI	P.mirabilis
2019	4.19	3.24-5.41	1335	XDR	Non-recurrent UTI	P.mirabilis
2020	3.43	2.6-4.52	1427	XDR	Non-recurrent UTI	P.mirabilis
2015	3.27	2.01-5.27	490	XDR	Recurrent UTI	P.mirabilis
2016	4.82	3.25-7.09	498	XDR	Recurrent UTI	P.mirabilis
2017	5.64	3.89-8.1	479	XDR	Recurrent UTI	P.mirabilis
2018	7.67	5.48-10.66	417	XDR	Recurrent UTI	P.mirabilis
2019	7.58	5.6-10.17	528	XDR	Recurrent UTI	P.mirabilis
2020	7.82	6.07-10.03	716	XDR	Recurrent UTI	P.mirabilis
2015	0.62	0.34-1.12	1771	PDR	Total	P.mirabilis
2016	0.51	0.27-0.98	1759	PDR	Total	P.mirabilis
2017	1.01	0.63-1.59	1791	PDR	Total	P.mirabilis
2018	0.88	0.53-1.46	1699	PDR	Total	P.mirabilis
2019	1.13	0.74-1.72	1863	PDR	Total	P.mirabilis
2020	0.79	0.49-1.27	2143	PDR	Total	P.mirabilis
2015	0.78	0.42-1.45	1281	PDR	Non-recurrent UTI	P.mirabilis
2016	0.56	0.26-1.16	1261	PDR	Non-recurrent UTI	P.mirabilis
2017	0.69	0.36-1.31	1312	PDR	Non-recurrent UTI	P.mirabilis
2018	0.86	0.48-1.54	1282	PDR	Non-recurrent UTI	P.mirabilis
2019	0.90	0.51-1.58	1335	PDR	Non-recurrent UTI	P.mirabilis
2020	0.35	0.15-0.84	1427	PDR	Non-recurrent UTI	P.mirabilis
2015	0.20	0.03-1.44	490	PDR	Recurrent UTI	P.mirabilis
2016	0.40	0.1-1.59	498	PDR	Recurrent UTI	P.mirabilis
2017	1.88	0.98-3.57	479	PDR	Recurrent UTI	P.mirabilis
2018	0.96	0.36-2.53	417	PDR	Recurrent UTI	P.mirabilis

S7 : Multiple drug resistance in *Proteus mirabilis* per year from 2015 to 2020

Year	Proportion	95% CI	N tested	Multiple resistance	Stratified	Pathogen
2019	1.70	0.89-3.25	528	PDR	Recurrent UTI	P.mirabilis
2020	1.68	0.95-2.93	716	PDR	Recurrent UTI	P.mirabilis
